# HOPE Asia Network Activity 2021—Collaboration and perspectives of Asia academic activity

**DOI:** 10.1111/jch.14215

**Published:** 2021-02-17

**Authors:** Kazuomi Kario

**Affiliations:** ^1^ Division of Cardiovascular Medicine Department of Medicine Jichi Medical University School of Medicine Tochigi Japan

At the beginning of 2020, none of us could have envisaged the events that would have come to define that year. The coronavirus disease‐2019 (COVID‐19) pandemic touched all corners of the globe and changed now billions of people lived. Travel restrictions and border closures saw the cancelation of major scientific meetings, while others went ahead using a “virtual” format. Academic collaborations that we previously took for granted suddenly became more difficult.

Although much research attention in 2020 has rightly been focused on COVID‐19, the importance of noncommunicable diseases (NCDs) such as hypertension must not be overlooked. The rapid spread of COVID‐19 around the world has impacted on the ability of countries to address and respond to NCDs.^1^ As well as disrupting health service provision, regular care, and preventive programs for NCDs such as hypertension, diabetes, and cancer have been interrupted.^1^ Taken together with pandemic‐related lifestyle changes, including lack of physical activity, stress, alcohol use, and unhealthy diet, the current lack of focus on NCDs has the potential to contribute to a different sort of pandemic in the coming years.

It was clear early on that subjects with pre‐existing NCDs were more likely to become severely ill or die from infection with SARS‐CoV‐2 virus.^2^ This immediate concern, combined with the importance of ensuring the continuity of NCD services, makes the goals and activities of the Hypertension Cardiovascular Outcome Prevention and Evidence in Asia (HOPE Asia) Network (Figure 1) even more relevant.

**FIGURE 1:**
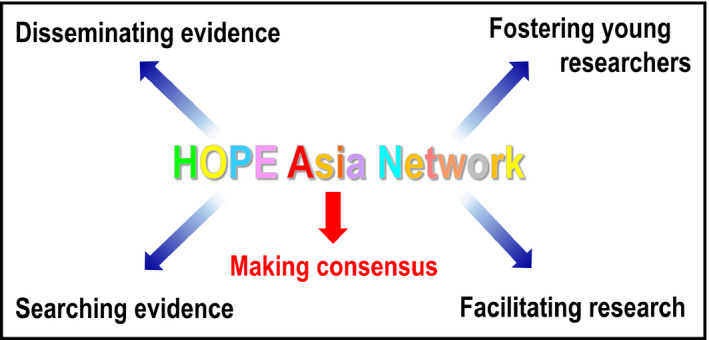
The five key missions of the HOPE Asia Network

## 1 HOPE ASIA NETWORK ACTIVITIES

Despite the challenging year, the HOPE Asia Network has continued to work toward the goal of improving the management of hypertension and organ protection to achieve “zero” cardiovascular events in Asia.^3^, ^4^ Specifically related to the pandemic, the group issued guidance on COVID‐19 and hypertension to help inform clinical management.^5^ In addition, the fruits of academic efforts under unparalleled circumstances are reflected in this issue. This includes several HOPE Asia Network documents. These cover practice points relating to implementation of ambulatory blood pressure (BP) monitoring in clinical practice, a review of mental health problems and hypertension in the elderly, the current status of adherence interventions for hypertension management, a comprehensive review of the association between hypertension and stroke in Asia, and discussion around the development of Asia‐specific tools for cardiovascular risk assessment. Measurement of BP in the clinic is the subject of another review, which highlights approaches to improve the ease and accuracy of this important parameter in subjects with hypertension.

## 2 ASIAN CHARACTERISTICS OF HYPERTENSION

Overall, the characteristics and consequences of hypertension in subjects from Asia differ in several important ways from those in Western populations. The clinical significance of nocturnal hypertension in Asia and the use of nocturnal home BP monitoring to improve patient management are discussed in this issue. There is also a closer look within Asia itself, highlighting ethnic differences in genetic variants, dietary choice, and lifestyle habits, along with sociodemographic differences, and variations in hypertension awareness and treatment control. Differences in culture, foods, and environment could contribute to different profiles of cardiovascular risk between Asian countries. This was highlighted by the results of a clinical study in this issue, which showed substantial differences in 24‐hour BP profiles even between neighboring countries in Asia (Japan and Thailand). In a study from Thailand, stroke was found to be the most common target organ damage in subjects with hypertensive emergency (consistent with stroke being a more common complication of hypertension in Asian subjects than coronary artery disease).^6^, ^7^

Isolated systolic hypertension (ISH) is the most common form of hypertension in the elderly and young adults. With rapid population aging in the Asia region, the prevalence of ISH will rise substantially. This highlights the importance of specific and locally‐relevant strategies to control ISH. The epidemiology and pathophysiology of ISH, along with risk factors, prognostic impact, and potential management strategies, are reviewed in this issue.

The impact of the change to BP thresholds in the latest American Heart Association (AHA) hypertension guidelines^8^ on the prevalence and management of hypertension has been the subject of much debate. The effects of using the different thresholds on BP control rates in Asia were determined in the HOPE Asia Network's Asia BP@Home study.^9^ In this issue, a study from South Korea investigated the impact of new AHA guideline threshold for office BP (decreased from ≥ 140/90 to ≥ 130/80 mmHg) on the correct identification of uncontrolled out‐of‐office BP.

## 3 LIFESTYLE AND DIET

One important feature of hypertension in Asian populations is high nighttime BP due to high salt sensitivity and salt intake.^4^, ^10^ This may be amenable to dietary modifications. Details of the evidence for dietary intervention for the management of hypertension in Asian populations are compared in a review article, which also contrasts dietary recommendations between the published guidelines from Western and Asian countries.

## 4 TREATING HYPERTENSION

The Asian characteristics of hypertension mean that some approaches to treatment might be more effective than others. Specific treatments reviewed in the current issue include the use of beta‐blockers in the region using data from the AsiaBP@Home study,^9^ the angiotensin receptor‐neprilysin inhibitor (sacubitril/valsartan), and the feasibility and utility of a polypill for cardiovascular disease prevention in Asian populations. Local clinical trial data on some alternatives to pharmacological therapy (foot reflexology and pursed‐lip breathing combined with number counting) provide some interesting options to consider.

## 5 COMPLICATIONS AND COMORBIDITIES

Hypertension is an important cardiovascular risk factor. A study presented in this issue showed that algorithms that rely on ICD‐10 diagnosis codes in combination with data on specific drugs and medical procedures appear to be valid for identifying major adverse cardiovascular events (MACE) in Japanese claims databases. This could be useful for predicting life‐threatening complications in subjects with hypertension. Other important comorbidities are also reviewed in this issue, including end‐stage kidney disease, obstructive sleep apnea, insulin resistance, and erectile dysfunction. Another review focusses on the pathophysiological relationships between long sleep duration, arterial stiffness and BP variability, and their effects on cardiovascular disease.

## 6 LATEST TECHNOLOGY

Newer information and communication‐based technology (ICT)‐based strategies provide different solutions to self‐monitoring of BP.^11^ The impact on COVID on the ability to conduct face‐to‐face consultations has highlighted the value of remote monitoring and telemedicine‐based strategies. These applications in the setting of natural disasters and the current pandemic are discussed in a review article in this issue. Another technology showing promising utility in hypertension is artificial intelligence (AI).^12^, ^13^ The advent of AI sheds light on new strategies for hypertension management, including insights from big data derived prediction. Tsoi and colleagues provide an overview of these revolutionary developments and their role in contributing to a future model for digital management of chronic diseases.

## 7 PERSPECTIVES

We are very happy to be able to present these local data and insights on hypertension and its management. The variety and quality of papers included in this issue goes to show that a global pandemic cannot destroy fruitful academic activity.

## CONFLICT OF INTEREST

None.
